# Nutritional Quality of Wholegrain Cereal-Based Products Sold on the Italian Market: Data from the FLIP Study

**DOI:** 10.3390/nu14040798

**Published:** 2022-02-14

**Authors:** Margherita Dall’Asta, Donato Angelino, Gaetana Paolella, Rossella Dodi, Nicoletta Pellegrini, Daniela Martini

**Affiliations:** 1 Department of Animal Science, Food and Nutrition, Università Cattolica del Sacro Cuore, 29100 Piacenza, Italy; margherita.dallasta@unicatt.it (M.D.); rossella.dodi@unicatt.it (R.D.); 2 Faculty of Bioscience and Technology for Food, Agriculture and Environment, University of Teramo, 64100 Teramo, Italy; dangelino@unite.it; 3 Department of Chemistry and Biology “A. Zambelli”, University of Salerno, 84084 Fisciano, Italy; gpaolella@unisa.it; 4 Department of Agricultural, Food, Environmental and Animal Sciences, University of Udine, 33100 Udine, Italy; 5 Department of Food, Environmental and Nutritional Sciences (DeFENS), Università degli Studi di Milano, 20133 Milan, Italy; daniela.martini@unimi.it

**Keywords:** cereals, fibre, nutrition claim, health claim, nutrition declaration, food labelling

## Abstract

The consumption of wholegrains (WG) is encouraged worldwide, but the lack of a common legal definition of such products leads to an unclear classification and identification on the grocery store shelf. In Italy, several products are generally sold as WG, but it cannot be determined if they are made entirely with all WG cereal(s) or if they are partially produced with WG ingredients (PWG). The aims of this study were to (a) survey the number of cereal-based food items formulated with WG, PWG, or refined (RG) present on the Italian market; and (b) analyse the nutritional quality, intended as nutrition facts, of WG products in comparison to PWG and RG. Nutritional information and declarations were retrieved from packs of 3040 products belonging to five different categories: breakfast cereals, biscuits, sweet snacks, bread, and bread substitutes. A descriptive analysis of the products and comparison of energy, macronutrients, fibre and salt among RG, PWG and WG products within each category was performed. In all categories, a major portion of the products did not contain WG ingredients. Results showed that the nutritional quality of RG, PWG, and WG products varied in relation to the product category and that WG inclusion cannot be always considered a marker of the overall nutritional quality of foods. Instead, it is necessary to evaluate the global product characteristics, and it is important to pay attention to differences between WG and PWG products that can be perceived by consumers as equivalent.

## 1. Introduction

Several cereals are key ingredients of many of the foods consumed worldwide on a daily basis. These cereals have a common kernel structure and are composed of a starchy endosperm surrounding the germ and external hard outer layers called bran, which particularly rich in micronutrients and bioactive compounds other than fibre [1]. In agreement with many dietary guidelines, cereal-based products are staple foods that should provide the major part of the daily calorie intake [2]. This energy is mainly due to the high content of complex carbohydrates and to a discrete amount of proteins. As mentioned, there is a notable presence of fibre and other micronutrients and bioactives concentrated in the bran layer which is almost totally removed during the milling process, thus resulting in much higher amounts in wholegrain (WG) cereal-based products than in the refined ones [3,4].

As a result of this different nutritional composition, dietary patterns rich in WG and WG-based products have different effects on nutritional status and health outcomes compared to the ones including refined cereal [5]. For instance, consumption of WG in adults was associated with significantly higher daily intakes of dietary fibre and several vitamins (thiamine, riboflavin, vitamin B6) and minerals (iron, calcium, potassium, phosphorus, zinc, magnesium) that are abundant in these products, compared to those who did not consume them [6]. Moreover, it is well-established that a high consumption of WG products is associated with a lower risk of non-communicable diseases, such as cardiovascular diseases [7,8,9], colorectal cancer [10], type-2 diabetes [11], symptoms of metabolic syndrome [12], and to a lower mortality rate for several causes [13,14]. Moreover, WG cereals represent one of the key foods at the base of the Mediterranean Diet pyramid, which therefore should be included daily and preferred to their refined counterparts [15]. For all these reasons, dietary guidelines worldwide suggest an increase in the consumption of WG, even though quantitative recommendations of WG are not clearly defined and/or consistent among the countries [16]. This last aspect may be also attributable to a lack of a legally binding definition of WG and, in turn, of WG flour and products [16]. According to the European Union’s agricultural legislation, WGs are “grains from which only the part of the end has been removed, irrespective of characteristics produced at each stage of milling [17]”. The European Food Safety Authority supports the definition of the American Association of Cereal Chemists, which states that WGs “consist of the intact, ground, cracked or flaked caryopsis, whose principal anatomical components—the starchy endosperm, germ and bran—are present in the same relative proportions as they exist in the intact caryopsis”, while EU-sponsored HEALTHGRAIN forum agreed that “whole grains shall consist of the intact, ground, cracked or flaked kernel after the removal of inedible parts such as the hull and husk [3]”. The definition of WG products is even more complex and not consistent across countries. For instance, wholegrain products in the UK and USA must include ≥51% of WG ingredients (on a wet matter basis). In European countries, such as Sweden and Denmark, WG ingredients must be ≥50% (on a dry matter basis) in WG products, while in Germany, WG bread must contain 90% WG [18]. The one recommended by the HEALTHGRAIN forum is that a WG food should contain “at least 30% whole-grain ingredients in the overall product and more whole grain than refined grain, both on a dry weight basis [16,19]”. Because of this uncertain and arbitrary definition for WG products, only a few countries and health-promoting organizations around the world defined and approved food labeling criteria and health claims on WG and WG products, but this was not done unanimously [20]. This leads to the presence on the market of products labelled as WG but meeting different requirements that vary from country to country. As a result, in Italy and Europe, a wide range of foods are sold as WG products or as products containing at least one WG cereal within the ingredients, which can therefore differ for the number and the amount of WG cereal constituting the product. Moreover, just the presence of WG may allow the consumer to perceive that these foods healthier than the ones without WG, independent of their content of WG. Besides that, previous investigations have shown that a large amount of the population is still not aware of the health benefits of WG [21,22]. Moreover, it is well-known that the overall nutritional quality of a food product is the result of many different aspects, including but not limited to the energy, macro and micronutrient content, as reported in the mandatory nutrition declaration in agreement with the European Union Regulation n.1169/2011 [23].

In this regard, even if WG products are supposed to be healthier than refined ones, the overall nutritional quality of commercially prepacked WG products sold on the Italian market have been barely investigated, and the hypothesis of the presence of the WG claim as a proxy of the total nutritional quality of the product has not been verified. Therefore, the aims of the present work were (a) to provide a descriptive analysis of WG products present on the Italian market, and (b) to investigate the overall nutritional quality of such products in comparison with products either only partially formulated with WG cereals or completely refined in nature.

## 2. Materials and Methods

### 2.1. Data Collection

Data were collected from the online surveys conducted in previous studies of the same project [24,25,26] and updated in June 2021. Prepacked cereal-based products considered in the present study were selected from the major Italian retailers on a home-shopping website (Bennet, Carrefour, Conad, Coop Italia, Crai, Despar, Esselunga, Il Gigante, Iper, Pam Panorama, Selex, Sidis). Products were considered eligible for the study if belonging to the following food categories: breakfast cereals, cereal-based sweet snacks, biscuits, bread, and bread substitutes. All the prepacked foods for which mandatory product information must be included directly on the package or on a label attached, as stated in the European Union Regulation n.1169/2011 were included as eligible products. Conversely, foods excluded after the online search were: (i) not prepackaged foods; (ii) items with incomplete images on all of the sides of the packaging; (iii) unclear images of information required; (iv) and products that were marked as ‘product currently unavailable’ on all the online stores selected during the whole data collection period.

### 2.2. Data Extraction

The complete images of all the sides of the pack were analysed and all information was extracted for each eligible product. For each item, the quali-quantitative and specifically regulated [23] information was collected: company name, brand name, descriptive name, energy (kcal/100 g), total fat (g/100 g), saturates (g/100 g), carbohydrate (g/100 g), sugars (g/100 g), protein (g/100 g), salt (g/100 g), and fibre (g/100 g). For the samples without indication of fibre content (since it is not mandatory according to Regulation n.1169/2011 [23]), this was calculated by subtracting the energy provided by each macronutrient (carbohydrates, protein and fats) from the total energy and dividing the resulting value by 2 kcal/g, which is the conversion factor for the calculation of energy as stated in the Regulation (EU) n. 1169/2011 [23]. All the ingredients reported in the “list of the ingredients” were extracted. Moreover, voluntary regulated declarations such as nutrition or health claims (NHC) as listed in the Regulation (EC) n.1924/2006 [27], gluten-free (GF) declarations (either ‘specifically formulated for celiacs’ or ‘containing gluten’) [28], and products declared as organic [29], were collected. The accuracy of the extracted data was double-checked by two researchers (MDA and DA), and inaccuracies were resolved through secondary extractions made by a third researcher (DM). A dataset was created with all the collected data, and items were sub-grouped for specific comparisons. By considering the descriptive name and the list of ingredients reported on the pack, items were labelled in detail as: (i) WG products when the product was defined as WG and all cereal-based ingredients in the list were defined as WG (e.g., “WG bread”); (ii) products partially formulated with WG ingredients (PWG), when one or more (but not all) cereal-based ingredients were WG (e.g., “breakfast cereals with WG wheat flakes”) and (iii) refined products (RG), when none of the ingredients was wholegrain.

### 2.3. Statistical Analysis

Data distribution was assessed using the Kolmogorov-Smirnov test. Data are expressed as a percentage or reported as median (interquartile range) for nutritional values. Differences in terms of energy, macronutrients, fibre and salt contents per 100 g of products for each item among WG, PWG and RG were analysed with Kruskal–Wallis non-parametric one-way ANOVA for independent samples with multiple pairwise comparisons. A Principal Component analysis (PCA) with varimax rotation was performed in order to evaluate the inter-product nutritional variability of products in terms of energy, macronutrients, salt and fibre contents per 100 g. In particular, score plots were organized to highlight product characteristics, i.e., category and presence/absence of wholegrains. Statistical analyses were carried out using IBM SPSS Statistics^®^ (Version 25.0, IBM corp., Chicago, IL, USA) and performed at *p* < 0.05 of significance level.

## 3. Results

### 3.1. Food Items Analysed

From a total of 3284 products initially retrieved, 244 products were excluded based on the exclusion criteria. In detail, among categories, “biscuits” was the one with the highest number of excluded products (18%), while in all other categories the % of exclusion ranged from 1 to 8%. As a result, a total of 3040 products were included in the analysis and grouped into the five different categories considered (“breakfast cereals”, “biscuits”, “sweet snacks”, “bread”, and “bread substitutes”). In Table 1, the number of items considered for each category of products, divided on the basis of the inclusion of WG (RG, PWG, WG), is reported. In all the categories, RG products prevailed on WG and PWG. WG products were more abundant than PWG in all categories, excepted for “breakfast cereals”.

Both WG products and products formulated with PWG ingredients represent a limited part of the total considered products: 24% for “breakfast cereals”, 17% “bread substitutes”, 17% for “bread”, 11% for “biscuits”, and 6% for “sweet snacks”. The categories with the highest % of WG products were “bread” (*n* = 52 out of 337), representing the 16% of products in this category, followed by “bread substitutes” (*n* = 120 out of 1016), representing the 12% of total samples. Conversely, the categories with the lowest number of WG items were “Sweet snacks” (*n* = 21 out of 510), corresponding to the 4% of the total category, “breakfast cereals”, and “biscuits”, with 31 and 57 items (8% and 7%), respectively.

For each category, the number of RG, PWG and WG products carrying at least one NHC, or boasting GF declarations, organic certification other than being brand or private label was calculated (Table 2). Among them, we found that few WG or PWG products carried specific declarations. In particular, a low number of products carried health claims in all categories, except the “breakfast cereals” category (14% and 34% of WG and PWG products, respectively). Moreover, few WG products displayed GF declarations in all categories and no one product belonged to the “sweet snacks” category; concerning this latter category, no organic PWG products were found. On the contrary, many WG or PWG products boasted a nutrition claim, with the highest percentage in the “breakfast cereals” category (87% WG products and 84% PWG products).

The number of products carrying nutrition claims concerning fibre (“source of fibre” corresponding to ≥3 g/100 g or “rich in fibre” corresponding to ≥6 g/100 g), which can be related to WG inclusion, was analysed. Interestingly, the majority (i.e., >50% for all categories except for PWG “sweet snacks”) but not all WG and PWG items carried these claims on the packaging. A further analysis was conducted to quantify the number of products without a claim concerning fibre that were potentially eligible for this declaration. About 62% of products in total showing a content of fibre higher than 3 g/100 g were found to be potentially eligible to make a fibre-related claim, without presenting this nutrition claim on the packaging.

### 3.2. Nutritional Composition of WG, PWG, RG Products for Each Category

Considering the nutrition facts reported on the pack, differences among RG, PWG and WG products within each category were analysed (Table 3). PWG and WG products had a similar (*p* > 0.05) energy content for “Biscuits”, “Bread”, “Bread substitutes”, and “Sweet snacks”. Particularly, the latter was the only category in which WG and PWG products did not differ either from each other or from RG products. On the contrary, WG “Breakfast cereals” presented lower energy compared to RG and PWG products, which resulted in them being not significantly different each other. For total and saturated fats, differences among RG, WG and PWG were not consistent among categories. “Biscuits” and “Bread substitutes” were the only categories showing a lower fat content for WG products than RG, despite that for “bread substitutes” a similar fat content was found for WG and PWG items. Total carbohydrate content was similar for WG and PWG products in the “biscuits”, “sweet snacks” and “bread” categories, while WG “bread substitutes” presented a lower carbohydrate content compared to both PWG and RG products. The protein content was higher in WG products for “breakfast cereals” and “bread substitutes” than in PWG and RG products, while for “biscuits”, “bread” and “Sweet snacks” categories a similar content of protein for WG and PWG was found (*p* > 0.05). The salt content was lower for RG “breakfast cereals”, “biscuits” and “sweet snacks” compared to the respective WG-containing products. Instead, the salt content was similar for RG, PWG, WG “bread”, and among “bread substitutes” was the highest in RG items and the lowest in the PWG ones (*p* < 0.05). The fibre content was similar among PWG and WG products, and higher with respect to the RG ones (*p* < 0.05) in most categories. The only exceptions were registered for “breakfast cereals” in which PWG products presented a similar fibre content to RG, and for “bread substitutes” in which PWG was lower in fibre than WG but higher than RG products.

### 3.3. Inter-Product Variability of the Nutritional Composition of Products in Analysed Categories

**Figure 1:**
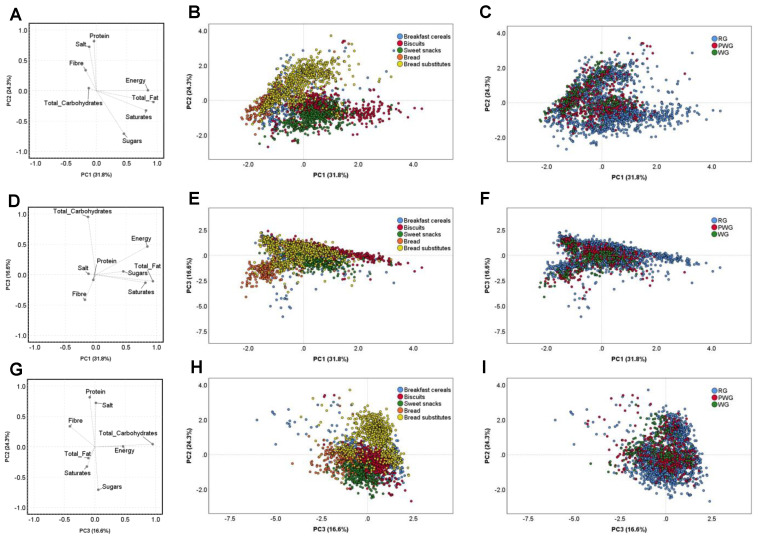
Principal component analysis (PCA) describing the inter-product variability based on the nutritional composition. Loading plots of PC1 versus PC2 (**A**), PC 1 vs. PC3 (**D**), PC3 vs. PC2 (**G**) are showed by considering energy (kcal/100 g), total fat (g/100 g), saturates (g/100 g), carbohydrate (g/100 g), sugars (g/100 g), protein (g/100 g), salt (g/100 g), and fibre (/100 g). Score plots of the nutrient composition of items are shown by considering the categories of products (**B**,**E**,**H**) and the classification by WG presence (RG, PWG, WG) (**C**,**F**,**I**). RG: refined grain; PWG: partially produced with wholegrain; WG: wholegrain.

The variability in the nutritional composition of the considered products was deepened by means of a PCA, as shown in Figure 1. On the whole, the PCA explained 72.7% of the total variability, but with the need for three main PCs. PC1, describing the 31.8% of the total variability, was positively loaded by energy, total and saturated fats and sugars, while PC2—24.3% of the variability, was positively loaded by protein, fibre and salt. Lastly, PC3—16.6% of the variability—was positively loaded by total carbohydrates, energy and sugars, while negatively loaded by total fats and saturates (Figure 1A,D,G). Concerning the product variability (Figure 1B,E,H) some categories may be defined on the basis of their nutritional content: bread substitutes were mainly described by high energy, total carbohydrate and salt contents, while sweet snacks were high in total fat, saturates, and sugars. When the presence of wholegrains in the product was taken into account, it was not possible to cluster RG products from those presenting wholegrains in terms of nutritional profile. Again, no grouping was demonstrated between the PWG and WG types of products in terms of energy, nutrients, fibre or salt (Figure 1C,F,I).

## 4. Discussion

To the best of our knowledge, this is the first survey focusing on prepacked wholegrain cereal-based products sold on the Italian market by dividing items into wholegrains, with at least one wholegrain ingredient (partially produced with wholegrains), and refined products. In particular, this study gives an overview of the characteristics and nutritional composition of more than three thousand products from several food categories, such as “breakfast cereals”, “biscuits”, “sweet snacks”, “bread”, and “bread substitutes”, as well as to the mandatory and voluntary information reported on the packaging of products.

Overall, and as expected, the main load of prepacked products considered in this survey was prepared with refined cereals (in the range 76–94% corresponding to “breakfast cereals” and “sweet snacks”, respectively). These data reflect the scarce demand and in turn the low consumption of WG-formulated products by Italian consumers, as previously reported [30]. In fact, data from the Italian Nutrition and Health Survey carried out between 2010 and 2013 described a regular consumption of WG products (more than once per week) in only 26.7% of adults, with bread representing one of the main sources of WG for the Italian population [30]. In the present survey, the “Bread” category presented the highest number of products prepared with 100% WG cereals (16%), even though this percentage described well the low presence of WG products on the shelf, which should be increased to promote the consumption of wholegrain products. Many strategies for promoting WG intake have been proposed, such as to increase the availability of WG products on the market, to ameliorate sensory properties, to reduce product costs, to gradually increase the “exposure” of consumers to these products, and finally to improve labelling for a clear identification of WG products [31]. As already mentioned, it is noteworthy that, despite the ascertained health benefits [5,19], the consumption of WG products is still lower than recommended. In 2010, the Global Dietary Database examined the wholegrain intake in 28 EU countries [32]. Considering the adult populations, the mean wholegrain intake in Italy was 11 g/day for both males and females, in Germany it was ~120 g/day and in France it was 36 g/day for both males and females. In Italy, in particular, the percentage of WG consumers from 2010–2013 appears to be quite low and still below that recorded in other countries of Europe, where consumption is frequently over 50% [30]. Interestingly, one of the main causes could be the scarce knowledge of WG products’ healthy benefits. Among adults, a greater consumption of WG was associated with a higher educational level and healthier lifestyle, including physical activity and the avoidance of smoking, while eating-related behaviours such as eating out of the home were inversely associated with wholegrain intake [30].

In this study, we compared the nutritional declaration of RG, PWG, and WG products for a better understanding of whether the WG presence in product formulation, as an exclusive source of cereals or as one of the product ingredients, can be a marker of product quality. In fact, it is important to reiterate that the lack of a shared legal definition of the WG product leads to a heterogeneous scenario in which WG products can strongly differ in their product formulation and nutritional characteristics. Therefore, in Italy and in Europe, due to a lack of regulation on the percentage of WG cereal that has to be included for the “claimed” WG product, it is interesting to comprehend whether nutritional characteristics of products only partially formulated with WG ingredients may have a nutritional profile similar to that of 100% WG products. By comparing the nutritional characteristics, WG-formulated products (both WG and PWG) presented differences and similarities that were not consistent among categories. In general, as expected the fibre content was the lowest in RG products but did not differ between PWG and WG, except for “breakfast cereals” and “bread substitutes”, in which PWG products contain less fibre than their WG counterpart. Interestingly, not all WG and PWG products presented a fibre content sufficient for bearing a fibre claim on the package. Energy, macronutrients and salt varied across WG, PWG and RG products depending on the category. For instance, WG and PWG “sweet snacks” and “biscuits” presented a similar nutritional profile, except for simple sugars, which were lower only in WG products. Interestingly, the salt content, whose consumption represents an urgent nutritional issue in Italy [33] and worldwide [34], proved to be higher in both PWG and WG sweet products (i.e., breakfast cereals”, “sweet snacks” and “biscuits”) than their RG counterparts. Bitterness is one of the key sensory attributes known to restrict the use of products containing WG. Several approaches have been proposed to mask the bitter taste in WG products, and one of them is the addition of salt [35]. Bakke et al. [36] successfully demonstrated that the bitterness of three green vegetables was reduced by adding salt without changing other attributes such as aroma or texture. This finding highlights even more the need to pay attention to the whole nutrition characteristics of products.

The present study showed that, likely due to the lack of a non-binding definition, WG products cannot be considered a marker of product quality a priori, but it is necessary to take into account the overall nutritional quality of products, especially for some product categories. Similar results have been recently underlined within the same project (FLIP project) in which the authors also stated that fibre-related nutrition claims, which can in some cases also be related to WG products, should not be considered as a marker of a better nutritional profile for breakfast cereals [37]. Other studies from the same project already highlighted the need to consider the global quality of the products instead of declarations about the general characteristics of products which may be misleading [24,26,38].

To help consumers in clearly identifying WG products, it would be desirable to reach a clear definition of WG products that is shared by the scientific community worldwide. In our opinion, cereal-based WG products should be produced only with WG ingredients, as is done in the WG category considered in this survey, and they should contain a relevant amount of WG ingredients in the products as recently proposed [39]. This would avoid providing misleading information to consumers and will likely promote WG consumption and in turn increase fiber intake.

This study clearly demonstrated that the nutritional profile of PWG and WG is strictly dependent on the category of product. In fact, PWG products generally showed similar characteristics to their 100% WG counterparts, but there are some cases where PWG were more similar to refined products. Certainly, due to a lack of a clear WG product definition at the national level, it is difficult to investigate the best criterion for classifying and fixing the standard of quality of WG products. As a consequence, it is challenging to promote WG product consumption through correct and regulated declarations on the packaging and therefore to correctly help consumers during a purchase. Previously, other authors underlined the need to find the best criteria for WG product classification and for supporting consumers in identifying the WG products with the best nutritional characteristics during purchase [40]. This issue is even more pronounced for products formulated with one or more, but not all, WG cereals, which can be perceived by consumers as being as healthy as 100% WG cereal-based products even if presenting a nutritional characteristic more similar to refined products. This phenomenon is well described as the “halo effect” [41], which induces consumers to “assign” to foods a healthy value in relation to their characteristics reported on the packaging. Certainly, labelling foods as “wholegrain” may be the right way to promote the consumption of WG products among consumers and to increase the overall quality of the diet. In this regard, Marinangeli et al. highlighted that labelling WG is one of the multiple opportunities to use labelling to promote the consumption of quality carbohydrate-rich foods, together with focusing on the low glycaemic index and glycaemic response claims or boasting dietary fibre nutrient content claims and associated dietary fibre-based health claims [42]. Thus, initiatives aimed at helping consumers in reading and understanding information reported on the food labeling would be useful to help them in making conscious food choices, which is also related to the selection of WG products.

An additional tool to promote WG consumption is through international and national dietary guidelines. As already pointed out, dietary guidelines include a variety of starchy foods in wholegrain form [2] and only a few countries in the European Union have adopted a quantitative recommendation on wholegrain intake. The Italian Dietary Guidelines for Healthy Eating suggests a preference for foods naturally rich in dietary fibre, such as whole cereals, pulses, fruit and vegetables, without establishing a specific quantitative indication for the consumption of WG products [43], which could help in reaching the daily reference intake for fibre of 12.6–16.7 g/1000 kcal for adults and 8.4 g/1000 kcal in childhood, and the suggested dietary target for pathology prevention fixed at 25 g/day of fibre [44].

The present study has several strengths and limitations worthy of mention. Among the former, this is the first study investigating the characteristics and the nutrition facts of a wide large of WG and not WG products currently on the market, including almost all categories of cereal-based products. Secondly, by collecting information on the food labelling, we were able to simultaneously focus on the nutrition declaration but also on other aspects (i.e., brand, NHC, organic and gluten-free declaration) which may be of interest for the consumers. Among limitations, it is noteworthy that the information reported on the pack does not allow, for instance, for the inclusion of some nutrients not mandatory in the nutrition declaration, such as micronutrients, which may be higher in WG compared to not WG products. Moreover, it was not possible to take into account other characteristics of products, such as the quality of the starting ingredients, which may be rich, for instance, in other components not accounted for in the nutritional declaration, but potentially abundant in WG products (e.g., bioactive compounds). Lastly, although a large part of the products have been collected through the home shopping websites of the major retailers, not all food items present on the market have likely been evaluated. Finally, since a barrier to WG product purchase can be the price, it would be interesting to investigate, in the future, differences among the cost of WG, PWG and RG products.

## 5. Conclusions

The results of the present study suggest that wholegrain products included in this survey have only limited beneficial nutritional characteristics than refined products, mainly in terms of fibre content and, only in some cases, to products partially formulated with wholegrain ingredients.

These data certainly support the lack of a need to consider a WG “declaration” on the pack as a synonym of a healthy product without taking into account the general quality of the products. Moreover, data highlight the need of introducing a legal definition of “wholegrain” that will avoid the current heterogeneity of products on the market, which also causes misunderstanding among consumers. This would promote a clear and regulated WG product formulation and labelling.

Reaching this goal would be useful to the food industry for reformulating food products with the purpose of improving the nutrition profile. Likewise, this will also be beneficial for consumers, who would be able to more easily identify WG products on the shelf. In turn, this will likely lead to increased consumption of these products and the reaching of dietary guideline goals that may positively influence dietary intake and human health.

## Figures and Tables

**Table 1:** Descriptive analysis of RG, PWG and WG products for each category.

WG Inclusion	Breakfast Cereals	Biscuits	Sweet Snacks	Bread	Bread Substitutes
RG (%)	289 (76%)	708 (89%)	478 (94%)	281 (83%)	843 (83%)
PWG (%)	62 (16%)	33 (4%)	11 (2%)	4 (1%)	51 (5%)
WG (%)	31 (8%)	57 (7%)	21 (4%)	52 (16%)	119 (12%)
Total	382	798	510	337	1013

RG: refined grain; PWG: partially produced with wholegrain; WG: wholegrain. Total is the sum of all the products in each category.

**Table 2:** Number of products in each category in relation to regulated declarations reported on the packaging and brand of products.

		Breakfast Cereals			Biscuits			Sweet Snacks			Bread			Bread Substitutes		
WG Inclusion		RG	PWG	WG	RG	PWG	WG	RG	PWG	WG	RG	PWG	WG	RG	PWG	WG
Nutrition claim	No	101	10	4	549	14	21	415	7	6	219	1	24	607	19	39
	Yes	197	52	27	159	19	36	63	4	15	62	3	28	238	32	81
	%	66	84	87	22	58	63	13	36	71	22	75	54	28	63	68
Nutrition claim on fiber	No	167	20	12	627	15	25	460	7	7	225	1	25	730	24	43
	Yes	122	42	19	81	18	32	18	4	14	56	3	27	113	27	76
	%	42	68	61	11	55	56	4	36	67	20	75	52	13	53	64
Health claim	No	257	41	20	704	32	54	475	11	20	281	4	50	804	51	113
	Yes	41	21	11	4	1	3	3	0	1	0	0	2	41	0	7
	%	14	34	35	1	3	5	1	0	5	0	0	4	5	0	6
Organic	No	210	59	21	646	22	41	450	11	15	246	3	45	622	33	89
	Yes	88	3	10	62	11	16	28	0	6	35	1	7	223	18	31
	%	30	5	32	9	33	28	6	0	29	12	25	13	26	35	26
Gluten free	No	269	62	30	661	32	56	446	9	21	251	3	50	684	35	115
	Yes	29	0	1	47	1	1	32	2	0	30	1	2	161	16	5
	%	10	0	3	7	3	2	7	18	0	11	25	4	19	31	4
Branded	No	139	35	13	276	10	20	220	5	9	117	1	23	316	21	50
	Yes	159	27	18	432	23	37	258	6	12	164	3	29	529	30	70
	%	53	44	58	61	70	65	54	55	57	58	75	56	63	59	58

RG: refined grain; PWG: partially produced with wholegrain; WG: wholegrain. %: percentage of product with the declaration (yes) or branded.

**Table 3:** Comparison of nutritional composition within each category of product analysed in relation to WG inclusion.

			Fats		Carbohydrates				
Category		Energy kcal/100 g	Total g/100 g	Saturated g/100 g	Total g/100 g	Sugars g/100 g	Fibre g/100 g	Protein g/100 g	Salt g/100 g
Breakfast cereals	RG	385 (371–425) a	5.1 (2.5–14.0)	1.3 (0.5–3.4)	69.0 (61.0–80.0)	20.0 (7.0–26.6)	5.6 (3.6–8.2) b	8.0 (7.0–10.0) b	0.4 (0.1–0.8) b
	PWG	388 (379–406) a	5.8 (1.8–10.1)	2.2 (0.5–3.8)	74.5 (64.0–79.0)	19.0 (15.0–24.1)	5.5 (4.2–8.0) b	8.6 (7.0–9.5) b	0.6 (0.3–0.9) a
	WG	374 (367–385) b	2.9 (2.1–7.4)	0.8 (0.5–1.8)	68.0 (62.1–74.8)	16.0 (2.5–22.3)	7.4 (5.8–10.0) a	9.4 (8.4–13.0) a	0.7 (0.2–0.8) ab
Biscuits	RG	473 (451–493) a	19.0 (15.7–22.4) a	5.8 (2.4–11.0) a	67.0 (62.7–71.) a	24.0 (21.0–29.0) a	2.7 (2–3.5) b	7.2 (6.3–7.9) b	0.5 (0.3–0.7) b
	PWG	463 (445–473) b	18.0 (16.0–19.5) a	4.3 (2.2–6.7) ab	63.0 (61.4–67.) b	23.3 (19.0–26.0) b	6.0 (4.3–6.7) a	7.6 (7.5–8.8) a	0.6 (0.5–0.7) a
	WG	453 (445–467) b	17.0 (16.0–19.5) b	2.4 (1.9–4.4) b	64.0 (61.4–66.0) b	19.2 (18.0–24.0) c	6.7 (5.7–8.2) a	8.3 (7.7–9.0) a	0.6 (0.5–0.7) a
Sweet snacks	RG	408 (388–427)	18.7 (15.0–21.4)	6.8 (3.8–9.9)	53.3 (50.5–57.0) a	28.3 (22.0–34.0) a	1.8 (1.4–2.5) b	6.2 (5.5–7.1) b	0.5 (0.4–0.7) b
	PWG	410 (395–414)	19.0 (18.0–20.2)	3.5 (2.8–8.2)	49.0 (45.0–50.3) b	27.0 (24.0–29.0) a	3.4 (2.6–3.6) a	8.0 (5.2–10.4) a	0.7 (0.3–0.9) a
	WG	405 (378–429)	17.7 (15.0–21.0)	6.1 (2.9–8.9)	47.0 (46.0–50.0) b	19.7 (14.8–25.8) b	4.1 (3.5–6.0) a	8.3 (6.8–8.6) a	0.7 (0.5–0.7) a
Bread	RG	276 (259–290) a	4.5 (3.5–5.7) a	0.7 (0.5–1.0) a	48.6 (45.2–51.0) a	4.6 (3.2–6.2) a	3.0 (2.6–4.10) b	8.5 (7.9–9.5)	1.3 (1.1–1.4)
	PWG	228 (208–289) b	1.9 (1.5–2.7) b	0.5 (0.3–0.9) ab	40.5 (38.5–53.5) ab	1.6 (1.2–1.8) b	5.5 (4.8–6.6) a	7.6 (6.4–10.2)	1.2 (1.0–1.7)
	WG	259 (243–268) b	4.3 (2.0–5.2) a	0.6 (0.4–0.8) b	42.0 (40.3–44.3) b	4.0 (3.0–5.3) a	6.3 (5.0–7.9) a	8.8 (8.1–9.6)	1.3 (1.1–1.4)
Bread substitutes	RG	417 (380–443) a	10.3 (6.6–14.5) a	1.7 (1.0–3.0) a	68.0 (62.5–73.0) a	1.9 (1.1–3.0) b	3.1 (2.3–4.0) c	10.0 (8.0–11.7) b	1.8 (1.0–2.2) a
	PWG	393 (383–424) b	7.0 (3.3–10.1) b	1.0 (0.6–1.7) b	70.0 (66.0–74.5) a	2.0 (0.7–6.5) ab	6.0 (3.8–6.9) b	11.0 (8.2–12.0) b	1.4 (0.5–1.7) c
	WG	393 (376–413) b	8.0 (5.8–10.3) b	1.1 (0.8–1.6) b	64.2 (61.0–68.0) b	2.0 (1.5–4.0) a	7.5 (6–10.0) a	11.4 (10.0–13.0) a	1.5 (1.2–2.0) b

Values are expressed as median (25th–75th percentile). For each category, different letters in the same column indicate significant differences among RG, PWG, and WG (Kruskal–Wallis non-parametric one-way ANOVA for independent samples with multiple pairwise comparisons), *p* < 0.05. RG: refined grain; PWG: partially produced with wholegrain; WG: wholegrain.
